# Effectiveness of Combined Face-to-Face and Mobile-Based Motivational Interviewing on Oral Hygiene Status and Behavior Among Adolescents in Gorontalo, Indonesia: A Quasi-Experimental Study

**DOI:** 10.3390/dj14050316

**Published:** 2026-05-21

**Authors:** Deliyana Imelda Katili, Ayub Irmadani Anwar, Ichlas Nanang Afandi, Irfan Sugianto, A. Arsunan Arsin, Indra Fajarwati Ibnu, Nurlindah Hamrun, Irwan Irwan

**Affiliations:** 1 Faculty of Dentistry, Hasanuddin University, Makassar 90245, Indonesia; 2 Department of Dental Public Health, Faculty of Dentistry, Hasanuddin University, Makassar 90245, Indonesia; ayubanwar_mks@yahoo.com; 3 Department of Psychology, Faculty of Medicine, Hasanuddin University, Makassar 90245, Indonesia; ichlas.afandi@med.unhas.ac.id; 4 Department of Oral and Maxillofacial Radiology, Faculty of Dentistry, Hasanuddin University, Makassar 90245, Indonesia; sugiantoirfan@gmail.com; 5 Department of Epidemiology, Faculty of Public Health, Hasanuddin University, Makassar 90245, Indonesia; arsunan_arsin@yahoo.co.id; 6 Department of Health Promotion and Science Behaviour, Faculty of Public Health, Hasanuddin University, Makassar 90245, Indonesia; indra5462@gmail.com; 7 Department of Oral Biology, Faculty of Dentistry, Hasanuddin University, Makassar 90245, Indonesia; lindahamrun@unhas.ac.id; 8 Department of Public Health, Faculty of Sport and Health, Gorontalo State University, Gorontalo 96128, Indonesia; irwan@ung.ac.id

**Keywords:** motivational interviewing, OHI-S, toothbrushing behavior, adolescents, mobile health

## Abstract

**Background/Objectives:** Oral health problems among adolescents in Indonesia remain high, particularly in Gorontalo City. Conventional education approaches are often insufficient to promote sustained behavioral change. This study aimed to evaluate the effectiveness of combined face-to-face and mobile-based Motivational Interviewing (MI) on oral hygiene status (OHI-S), knowledge, and toothbrushing behavior among adolescents. **Methods:** A quasi-experimental study with a non-randomized control group and repeated measurements was conducted among 100 adolescents aged 15–18 years in Gorontalo, Indonesia. Participants were allocated into a combined MI group (face-to-face plus mobile application) and a face-to-face MI group. Knowledge was assessed using a validated 15-item questionnaire, and toothbrushing behavior was measured using a 24-item questionnaire; both instruments used dichotomous scoring and demonstrated good reliability (Cronbach’s alpha = 0.82 and 0.85, respectively). Oral hygiene status was evaluated clinically using the Simplified Oral Hygiene Index (OHI-S) by a calibrated examiner (κ = 0.82). Outcomes were measured at baseline and at three follow-up points over three months. Data were analyzed using the Wilcoxon signed-rank test and Mann–Whitney U test, with significance set at *p* < 0.05. **Results:** Within-group analyses showed improvements across all outcomes in both groups (*p* < 0.05). The combined MI group demonstrated greater improvements compared to the face-to-face group, with median knowledge scores increasing from 4 to 8 versus 5 to 7 (between-group *p* = 0.002), toothbrushing behavior from 10 to 15 versus 12 to 13 (*p* = 0.001), and OHI-S scores decreasing from 3.2 to 1.4 versus 2.8 to 2.0 (*p* < 0.001). These findings indicate a greater magnitude of change in the combined intervention group. **Conclusions:** The combined face-to-face and mobile-based MI approach was associated with greater improvements in oral hygiene status, knowledge, and toothbrushing behavior among adolescents compared to face-to-face MI alone. However, due to the non-randomized design, the findings should be interpreted as associations rather than causal effects.

## 1. Introduction

Oral health is a crucial aspect of general health that significantly impacts an individual’s quality of life. A healthy mouth not only means being free from diseases such as oropharyngeal cancer, infections, and gum disease, but also supports vital functions such as chewing, speaking, and expressing emotions [1,2,3]. Research shows a close relationship between oral health and various systemic diseases, such as diabetes, digestive disorders, stroke, and cardiovascular disease [4,5].

In Indonesia, data from the 2018 Basic Health Research (Riskesdas) reported that the prevalence of dental caries reached more than 80%, with a mean Decayed, Missing, and Filled Teeth (DMF-T) index of 4.6 among the general population aged ≥12 years. This value exceeds the World Health Organization (WHO) target for oral health, indicating a substantial burden of dental disease at the population level [6,7]. This condition is exacerbated by low knowledge, limited access to dental health services, and poor dental care practices, especially among adolescents [8,9].

Dental caries and periodontal disease, which are common in adolescents, are generally caused by poor oral hygiene, leading to the accumulation of plaque containing pathogenic bacteria such as Streptococcus mutans and Lactobacillus, which can cause pain, infection, and even tooth loss [10,11]. Dental health education (DHE), a commonly implemented school-based oral health promotion program in Indonesia, has been widely used to improve knowledge and preventive practices. However, such approaches often remain didactic and may be insufficient to promote sustained behavioral change [12,13].

One approach currently considered effective is Motivational Interviewing (MI), a patient-centered counseling technique aimed at increasing intrinsic motivation to change health behaviors [14,15,16]. However, implementing conventional MI in schools and healthcare facilities still faces challenges, including limited time and resources and minimal participant involvement [17].

In Gorontalo Province, oral health problems among adolescents are reported to be higher than the national average. According to the 2018 Basic Health Research (Riskesdas), the prevalence of dental caries in Gorontalo reached approximately 84–86%, compared to the national prevalence of around 80%, indicating a substantial regional burden. These findings highlight the need for more effective and targeted oral health interventions in this population [7]. Conventional counseling, which is one-way and passive, is less effective in fostering sustainable healthy behaviors in digital youth who are closely connected to technology [18]. Therefore, more interactive and sustainable intervention strategies are needed.

Building on this need for new approaches, the rapid development of information technology, particularly mobile applications, offers new opportunities to deliver interactive, personalized, and continuous health promotion. Integrating MI with mobile-based platforms may strengthen motivation, enhance engagement, and promote more consistent oral health behaviors among adolescents [19,20,21,22].

However, while these technological advances show promise, evidence on the effectiveness of combined face-to-face and mobile-based Motivational Interviewing (MI) interventions remains limited, particularly in Indonesia. Most studies have evaluated conventional MI or digital interventions separately, with few examining their combined effects on behavioral and clinical oral health outcomes.

Therefore, this study aimed to evaluate the effectiveness of a combined face-to-face and mobile-based MI intervention on oral hygiene status (OHI-S), knowledge, and toothbrushing behavior among adolescents in Gorontalo City, Indonesia.

It was hypothesized that adolescents receiving the combined intervention would demonstrate greater improvements in oral hygiene status, knowledge, and toothbrushing behavior compared to those receiving face-to-face MI alone.

## 2. Materials and Methods

### 2.1. Study Design and Participants

This study employed a quasi-experimental design with a non-randomized control group and repeated measurements. The study was conducted from January to April 2025 in selected senior high schools in Gorontalo City, Indonesia.

Participants were adolescents aged 15–18 years recruited through school-based sampling. Inclusion criteria were: (1) students aged 15–18 years, (2) willingness to participate and provide informed consent, and (3) access to a smartphone to support the mobile-based intervention. Exclusion criteria included: (1) presence of systemic diseases affecting oral health and (2) absence during follow-up assessments.

A total of 100 participants were enrolled and allocated into two groups based on school clusters to minimize contamination: (1) a combined intervention group receiving face-to-face Motivational Interviewing (MI) and mobile-based support, and (2) a comparison group receiving face-to-face MI only. Randomization was not performed due to the clustering approach; therefore, the potential for selection bias cannot be excluded. However, both groups were recruited from comparable school environments within the same geographic area.

The sample size was calculated using a two-independent-means formula with a significance level of 0.05 and statistical power of 80%. Based on previous studies reporting a mean difference in OHI-S of 0.8–1.0 and a standard deviation of 1.2–1.4, the minimum required sample size was 45 participants per group. After adjusting for a 10% potential dropout rate, the final sample size was set at 50 participants per group (total *n* = 100). This calculation was verified using G*Power version 3.1.

### 2.2. Intervention Description

The intervention consisted of Motivational Interviewing (MI) delivered in two formats: face-to-face MI and a combination of face-to-face MI with a mobile application component.

The face-to-face MI sessions were conducted by licensed dentists and delivered directly in the school setting. Each session lasted approximately 30–45 min and was conducted weekly during the intervention period. The sessions focused on improving oral hygiene knowledge and toothbrushing behavior through personalized counseling, goal setting, and behavioral reinforcement.

#### 2.2.1. Combined MI Group

Participants in the combined intervention group received additional support through a smartphone-based application designed to reinforce MI principles. The application was accessible via Android-based devices and was developed specifically for this study to deliver structured oral health education and behavioral reinforcement.

The application included the following components:

(1) Educational modules on oral health and dental hygiene;
(2) Daily reminders for toothbrushing (twice per day);
(3) Motivational messages tailored to reinforce behavior change;
(4) Self-monitoring features allowing participants to record daily toothbrushing activities.

Automated messaging was rule-based and scheduled at predefined intervals, rather than using adaptive machine learning algorithms. Participants received reminders twice daily and motivational messages three times per week throughout the three-month intervention period.

Compliance with application use was monitored through self-reported logs and periodic checks during follow-up visits.

#### 2.2.2. Face-to-Face MI

Face-to-face MI sessions were delivered by trained dental professionals (dentists and dental nurses) who had received standardized training in Motivational Interviewing techniques prior to the study. Each session lasted approximately 30–45 min and was conducted weekly in the school setting over the intervention period.

The MI sessions followed a structured approach based on core MI principles, including:

(1) Engaging participants through empathetic communication;
(2) Focusing on oral hygiene behaviors;
(3) Evoking intrinsic motivation for behavior change;
(4) Planning actionable goals.

Techniques such as open-ended questions, affirmations, reflective listening, and summarizing (OARS) were consistently applied. Participants were encouraged to identify personal barriers to oral hygiene and to develop individualized strategies for improving toothbrushing practices.

#### 2.2.3. Intervention Timeline

The intervention was conducted over a period of three months (12 weeks). During this period, participants in both groups received weekly face-to-face Motivational Interviewing (MI) sessions, resulting in a total of 12 MI sessions per participant. Each session lasted approximately 30–45 min and was delivered in the school setting by trained dental professionals.

In addition to the weekly face-to-face sessions, participants in the combined intervention group received continuous support through a mobile application throughout the 12-week period. The application provided daily toothbrushing reminders (twice per day), motivational messages (three times per week), and self-monitoring features to reinforce behavior change between MI sessions.

Outcome assessments were conducted at four time points: baseline (T0), month 1 (T1), month 2 (T2), and month 3 (T3). These repeated measurements were used to evaluate changes in knowledge, toothbrushing behavior, and oral hygiene status over time.

### 2.3. Data Collection Instruments

Data were collected using structured and validated instruments to assess knowledge, toothbrushing behavior, and oral hygiene status.

Knowledge was measured using a 15-item questionnaire covering oral hygiene practices, causes of dental caries, and preventive strategies. Toothbrushing behavior was assessed using a 24-item questionnaire evaluating frequency, timing, and brushing technique. Responses for both instruments were scored dichotomously (1 = correct, 0 = incorrect), and total scores were calculated by summing item responses.

Content validity was assessed by experts in dental public health, and pilot testing was conducted prior to the study. Internal consistency reliability was confirmed using Cronbach’s alpha (knowledge: 0.82; behavior: 0.85), indicating good reliability.

Oral hygiene status was evaluated using the Simplified Oral Hygiene Index (OHI-S) through clinical examination conducted by a calibrated examiner following standardized procedures. OHI-S scores were categorized as good (0.0–1.2), fair (1.3–3.0), and poor (3.1–6.0). Intra-examiner reliability was assessed using Cohen’s kappa (κ = 0.82), indicating strong agreement.

### 2.4. Data Analysis

Data were analyzed using nonparametric tests because the distributions were nonnormal. Quantitative variables with normal distribution were presented as mean (standard deviation), whereas nonnormally distributed variables were presented as median (interquartile range). The Wilcoxon signed-rank test was used for within-group comparisons, and the Mann–Whitney U test for between-group comparisons. The level of significance was set at *p* < 0.05. Effect sizes (r) were calculated using the formula r = Z/√N, where N represents the total number of observations included in the analysis, to estimate the magnitude of differences. The questionnaires demonstrated good internal consistency, with Cronbach’s alpha values of 0.82 and 0.85. Clinical examination for oral hygiene status (OHI-S) was conducted by a calibrated examiner, and intra-examiner reliability was assessed using Cohen’s kappa, yielding a value of 0.82, indicating strong agreement.

Due to the non-randomized design and the relatively small sample sizes within each group, non-parametric tests were selected to ensure robust data analysis. Although repeated measurements were obtained, the analysis focused on pairwise comparisons between baseline and follow-up time points. A global repeated-measures test (e.g., Friedman test) was not applied, as the primary objective was to assess specific changes over time rather than overall trends. Future studies with larger sample sizes and randomized designs are recommended to apply more comprehensive longitudinal analytical approaches.

### 2.5. Ethical Considerations

This study received ethical approval from the Research Ethics Committee of the Faculty of Dentistry, Hasanuddin University, Indonesia (Approval No. 046/KEPK FKG-RSGMP UH/EE/XI/2024; 13 November 2024). This study was not registered as a clinical trial because it employed a quasi-experimental design and did not meet the criteria for mandatory clinical trial registration. Informed consent was obtained from all participants and their parents or guardians prior to data collection. This study was not registered as a clinical trial due to its quasi-experimental design.

## 3. Results

Improvements in knowledge, toothbrushing behavior, and OHI-S scores were observed in both groups across follow-up measurements. The analysis focused on pairwise comparisons using nonparametric tests to evaluate changes within and between groups at defined time points.

### 3.1. Baseline Characteristics of Adolescents

A total of 100 adolescents participated in this study, with 50 allocated to the combined MI group and 50 to the face-to-face MI group. The mean age of participants was 16.5 (1.0) years, and the majority were female (80%).

Baseline characteristics between the two groups were generally comparable, as shown in Table 1. There were no statistically significant differences in age, knowledge scores, toothbrushing behavior, or OHI-S scores (*p* > 0.05). However, slight differences in median values were observed, indicating that baseline equivalence cannot be fully ensured.

### 3.2. Analysis of Differences in Knowledge of MI Interventions Face-to-Face with a Combination (Face-to-Face and Application-Based)

Within-group analysis using the Wilcoxon signed-rank test showed a statistically significant increase in knowledge scores in both groups (*p* < 0.05), as presented in Table 2.

In the combined MI group, the median knowledge score increased from 4 to 8, representing a 100% improvement from baseline. In contrast, the face-to-face MI group showed an increase from 5 to 7, corresponding to a 40% improvement.

Between-group comparison demonstrated significantly higher post-intervention knowledge scores in the combined MI group (*p* < 0.05). The calculated effect size indicated a large effect in the combined group (r = 0.52) and a moderate effect in the face-to-face group (r = 0.34).

### 3.3. Analysis of Differences in Tooth Brushing Behavior (Tooth Brush Practice) Before and After Intervention

Both groups demonstrated statistically significant improvements in toothbrushing behavior following the intervention (*p* < 0.05), as shown in Table 3.

The combined MI group showed an increase in median scores from 10 to 15, reflecting a 50% improvement in toothbrushing behavior, with a very large effect size (r = 0.87). Meanwhile, the face-to-face MI group improved from 12 to 13, representing an approximate 8% increase, with a moderate-to-large effect size (r = 0.45).

These findings indicate that the combined intervention resulted in a substantially greater behavioral change compared to face-to-face MI alone.

### 3.4. Analysis of Differences in OHI-S Score Before and After Intervention

Improvements in OHI-S scores were observed in both groups, with a greater reduction in the combined MI group, as presented in Table 4.

The combined MI group demonstrated a decrease in median OHI-S scores from 3.2 to 1.4, representing a reduction of approximately 56%, with a very large effect size (r = 0.85). In comparison, the face-to-face MI group showed a reduction from 2.8 to 2.0, corresponding to a 29% improvement, with a moderate-to-large effect size (r = 0.49).

Between-group analysis showed statistically significant differences in OHI-S scores at follow-up (*p* < 0.05), favoring the combined intervention.

### 3.5. Between-Group Comparison of Knowledge, Toothbrushing Behavior, and OHI-S

Post-intervention comparisons using the Mann–Whitney U test revealed significantly better outcomes in the combined MI group across all measured variables (*p* < 0.05), as summarized in Table 5.

The combined MI group achieved higher median scores in knowledge (8 vs. 7) and toothbrushing behavior (15 vs. 13), as well as lower (better) OHI-S scores (1.4 vs. 2.0).

Effect size analysis indicated moderate-to-large effects across outcomes (knowledge: r = 0.44; toothbrushing behavior: r = 0.49; OHI-S: r = 0.52), suggesting that the addition of mobile-based MI support produced meaningful and clinically relevant improvements compared to face-to-face MI alone.

## 4. Discussion

### 4.1. Analysis of Differences in Knowledge of MI Interventions Face-to-Face with a Combination (Face-to-Face and Application-Based)

The findings of this study indicate that both intervention approaches were associated with improvements in participants’ knowledge; however, the magnitude of change was greater in the group receiving the combined face-to-face and application-based Motivational Interviewing (MI). This suggests that the addition of mobile-based support may enhance the effectiveness of MI in improving oral health knowledge among adolescents. The consistent increase in mean knowledge scores across measurement stages reflects the effectiveness of this approach in strengthening participants’ understanding [23,24]. This finding aligns with numerous studies that emphasize MI’s superiority, particularly when combined with interactive methods such as face-to-face sessions. Notably, Lundahl et al. (2010) highlighted in their meta-analysis that MI is highly effective across contexts, particularly when delivered in direct interaction, thereby optimally increasing participant motivation and engagement [25].

The greater improvement observed in the combined intervention group may be related to the continuous reinforcement provided by the mobile application, which complemented the interactive and personalized nature of face-to-face MI sessions. While face-to-face MI allows for direct communication, individualized feedback, and the development of intrinsic motivation, the application-based component may support repeated exposure to information, reminders, and self-monitoring, thereby facilitating better knowledge retention.

Furthermore, research by Baer et al. supports these findings, showing that face-to-face sessions not only increase knowledge but also strengthen participants’ engagement in the behavior change process. This research suggests that high engagement in face-to-face sessions fosters a more interactive discussion atmosphere, enabling participants to better understand the material presented and to ask questions or raise concerns directly. In contrast, although the group that received only the intervention without MI also showed an increase in knowledge, the magnitude of the change was much smaller [26]. These results reinforce the notion that while digital media can be an effective and flexible learning tool, the presence of a face-to-face component remains crucial for optimizing the knowledge-building process. Supporting this, previous studies have shown that digital-based Motivational Interviewing (MI) is effective; however, the intensity and quality of behavior change tend to be greater in interventions incorporating face-to-face interaction [27,28].

Overall, the findings of this study underscore the potential value of combining face-to-face and digital delivery modes in MI-based interventions. This combined approach was associated with improvements in knowledge and may also contribute to stronger participant engagement and motivation in the learning process. Therefore, to achieve optimal results, it is recommended that health education interventions or other behavioral training utilize a blended strategy, combining face-to-face sessions with reinforcement through digital media or during the process. This approach has been shown to be more effective than relying solely on one method, as supported by previous studies have reported that Motivational Interviewing (MI) interventions can improve knowledge and health-related behaviors. For example, a systematic review by Gao et al. (2014) found that MI-based interventions were associated with improvements in oral health outcomes, including increased oral hygiene knowledge and reduced plaque indices [29]. In addition, Ellingson et al. (2019) reported that integrating MI with digital support tools led to higher participant engagement and improved adherence to health-related behaviors compared to standard approaches [27].

### 4.2. Analysis of Differences in Tooth Brushing Behavior (Tooth Brush Practice) Before and After Intervention

The results of this study confirm that Motivational Interviewing (MI) interventions delivered in a combination of both face-to-face and application-based sessions have a significant impact on improving tooth-brushing behavior in adolescents. The significant increase in average behavior in this group indicates that intervention methods involving direct interaction and continuous reinforcement throughout the intervention process can encourage more optimal behavior change [30,31,32,33]. These findings are consistent with previous research demonstrating that Motivational Interviewing (MI), particularly when delivered in blended formats, may be effective in supporting health behavior change among adolescents, including oral hygiene practices [33].

Research by Freudenthal Bowen (2020) [34] in the *Journal of Dental Research* showed that adolescents who received in-person MI intervention with digital support or regular reinforcement tended to maintain behavioral changes better than those who received only traditional education. Face-to-face interactions provide facilitators with the opportunity to build more personal relationships, motivate participants, and tailor messages to individual needs, while reinforcement throughout the process helps ensure messages are remembered and implemented [35,36,37,38].

Meanwhile, the group receiving only face-to-face intervention without MI elements also showed an increase in toothbrushing behavior, though the magnitude of the change was smaller than that of the combined MI group. This suggests that face-to-face education is indeed effective in increasing knowledge and behavior, but not optimal in facilitating sustainable behavior change. A study in the *Journal of Medical Internet Research* (JMIR) also reported that the MI approach combined with other methods is superior in internalizing healthy behaviors, as it can increase participants’ self-efficacy and commitment to change [27].

Overall, these findings indicate that intervention strategies combining Motivational Interviewing (MI) with face-to-face and application-based approaches may be associated with improved establishment and maintenance of healthy behaviors in adolescents, particularly tooth brushing behavior. Integrating these two methods not only increases the effectiveness of the intervention but also provides a more holistic and sustainable learning experience for participants. Therefore, the combined implementation of MI is recommended for adolescent health education programs to achieve more significant and sustainable behavior change.

### 4.3. Analysis of Differences in OHI-S Status Before and After Intervention

These findings reinforce previous research showing that a multimodal approach to health interventions, particularly utilizing digital technology as a support, can increase the effectiveness of behavior change. Studies published in the *Journal of Medical Internet Research* (*JMIR*) and *Patient Education and Counseling* confirm that integrating MI with online support—such as app reinforcement, reminder messages, or online education—can extend the duration of intervention effects, improve information retention, and make it easier for participants to incorporate health practices into their daily lives [39,40,41,42,43]. Furthermore, the use of digital technology in health intervention programs facilitates more flexible and continuous access to information. This allows adolescents to receive ongoing motivation and reminders outside of face-to-face sessions, resulting in more consistent and sustainable behavior change [44,45,46,47]. In addition, prior studies have reported that adolescents tend to respond positively to interactive, technology-based approaches, which may enhance engagement and self-efficacy in maintaining oral hygiene behaviors [27].

Although no statistically significant baseline differences were identified, small variations in median scores were observed, particularly in knowledge, tooth-brushing behavior, and OHI-S scores. These differences may have influenced the magnitude of change observed after the intervention. Therefore, the findings should be interpreted with caution.

Overall, the results of this study emphasize the importance of adopting intervention strategies that integrate face-to-face and online methods in health behavior change efforts. This approach not only increases the effectiveness of interventions but also provides more adaptive and relevant experiences for adolescents in the digital age. Therefore, the combined use of MI can be recommended as an effective strategy in oral health education programs for adolescents, to encourage better and more sustainable tooth brushing behaviors.

### 4.4. Between-Group Comparison of Knowledge, Toothbrushing Behavior, and OHI-S Scores After Intervention

The between-group comparison showed that the combined face-to-face and mobile-based Motivational Interviewing (MI) intervention led to greater improvements in knowledge, toothbrushing, and oral hygiene status (OHI-S) than face-to-face MI alone. This suggests the mobile component enhances the intervention’s effectiveness.

One possible explanation is that digital support provides continuous reinforcement beyond the initial session. Face-to-face MI offers personalized, patient-centered communication to build motivation. The mobile component adds regular reminders and engagement, which are critical for maintaining behavior change. This is relevant among adolescents, who often engage with digital platforms daily.

The greater improvement in oral hygiene status shows that behavioral changes led to measurable clinical outcomes. However, interpret these findings with caution, as the non-randomized design may introduce bias. Other external factors influencing oral hygiene were not fully controlled.

### 4.5. Study Limitations

This study has several limitations that should be considered when interpreting the findings. First, the quasi-experimental design without randomization, including the allocation of participants to school clusters, introduces the potential for selection bias and limits the ability to establish causal relationships between the intervention and outcomes. Differences in underlying characteristics between groups may have influenced the results.

Second, the sample was drawn from a single geographic area, which may restrict the generalizability of the findings to adolescents in other regions or settings. Third, although multiple follow-up measurements were conducted, the study did not employ longitudinal analytical approaches to fully assess changes across time points, thereby limiting the ability to draw firm conclusions about sustained behavioral change. In addition, the relatively short follow-up period further restricts the evaluation of long-term effects.

Furthermore, the assessment of tooth-brushing behavior relied partly on self-reported measures, which may be subject to reporting bias. Finally, the individual contribution of each intervention component (face-to-face MI and application-based MI) could not be isolated in this study.

Therefore, the findings should be interpreted with caution. Future studies using randomized controlled designs, larger and more diverse samples, longer follow-up periods, and more robust longitudinal analytical methods are recommended to strengthen the evidence base.

## 5. Conclusions

This study found that a combined face-to-face and mobile-based Motivational Interviewing (MI) approach was associated with greater improvements in oral hygiene status (OHI-S), knowledge, and toothbrushing behavior among adolescents compared to face-to-face MI alone. The integration of digital support with face-to-face MI may enhance participant engagement and provide continuous reinforcement, which could contribute to improved behavioral outcomes. However, given the quasi-experimental design, the absence of randomization, and the limited follow-up period, these findings should be interpreted as associations rather than causal effects. Further research using randomized controlled designs, larger sample sizes, and longer follow-up periods is recommended to confirm these findings and to better understand the long-term impact of combined MI interventions.

## Figures and Tables

**Table 1:** Baseline characteristics of adolescents.

Variable	Combined MI (*n* = 50) Median (IQR)	Face-to-Face MI (*n* = 50) Median (IQR)	*p*-Value *
Age (years)	16 (15–17)	16 (15–17)	0.842
Knowledge score	4 (3–5)	5 (4–6)	0.091
Tooth-brushing behavior	10 (8–12)	12 (10–13)	0.073
OHI-S score	3.2 (2.4–3.8)	2.8 (2.0–3.5)	0.112

Sources: Primary data, 2025 * Mann–Whitney U test.

**Table 2:** Analysis of differences in knowledge before and after combined interventions.

Group	Pre-Test Median (IQR)	Post-Test 3 Median (IQR)	Z	*r*	*p*
Combined MI	4 (3–5)	8 (7–9)	−6.15	0.52	<0.001
Face-to-Face MI	5 (4–6)	7 (6–8)	−5.00	0.34	0.003

**Table 3:** Analysis of differences in tooth brushing behavior in adolescents who were given MI in combination (face-to-face and application-based) with MI given face-to-face.

Group	Pre-Test Median (IQR)	Post-Test 3 Median (IQR)	Z	*r*	*p*
Combined MI	10 (8–12)	15 (14–16)	−6.17	0.87	<0.001
Face-to-Face MI	12 (10–13)	13 (12–14)	−3.16	0.45	0.003

**Table 4:** Analysis of differences in tooth brushing behavior in adolescents who were given MI in combination (face-to-face and application-based) with MI given face-to-face.

Group	Pre-Test Median (IQR)	Post-Test 3 Median (IQR)	Z	*r*	*p*
Combined MI	3.2 (2.4–3.8)	1.4 (1.0–2.0)	−6.03	0.85	<0.001
Face-to-Face MI	2.8 (2.0–3.5)	2.0 (1.5–2.8)	−3.48	0.49	0.003

**Table 5:** Between-group comparison of knowledge, toothbrushing behavior, and OHI-S scores after intervention.

Variable	Group	Median (Post-Test)	Z Value	*p*	Effect Size (r)
Knowledge	Combined MI	8	−3.10	0.002 *	0.44
	Face-to-Face MI	7			
Toothbrushing Behavior	Combined MI	15	−3.50	0.001 *	0.49
	Face-to-Face MI	13			
OHI-S	Combined MI	1.4	−3.70	<0.001 *	0.52
	Face-to-Face MI	2.0			

* significant at *p* < 0.05. Mann–Whitney U Test.

## Data Availability

The data underlying this study cannot be made publicly available due to ethical considerations related to the protection of adolescent participants’ personal information. Anonymized data may be provided by the corresponding author upon reasonable request and subject to approval from the relevant ethics and institutional review boards.

## Abbreviations

The following abbreviations are used in this manuscript:

OHI-S	Oral Hygiene Index - Status
MI	Motivational Interview
